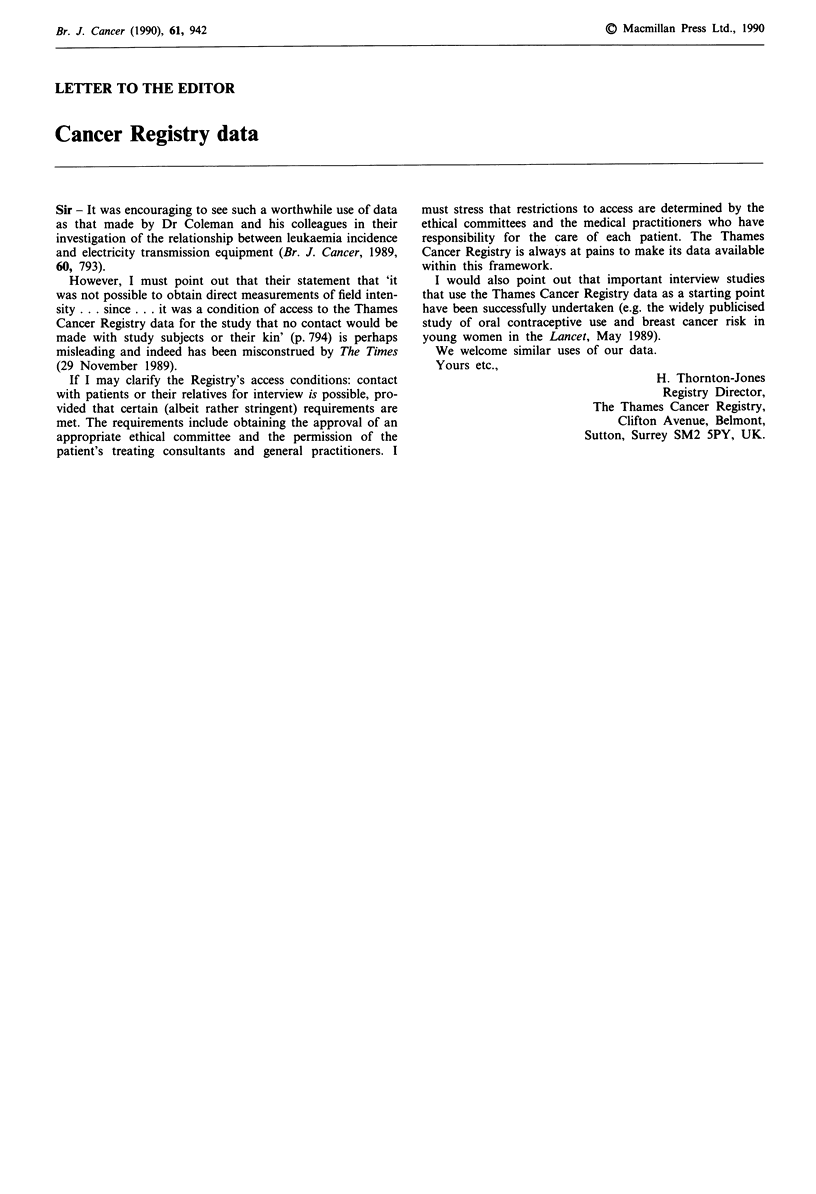# Cancer Registry data.

**DOI:** 10.1038/bjc.1990.211

**Published:** 1990-06

**Authors:** H. Thornton-Jones


 © Macmillan Press Ltd., 1990

### LETTER TO THE EDITOR

### Cancer Registry data

**Sir** – It was encouraging to see such a worthwhile use of data as that made by Dr Coleman and his colleagues in their investigation of the relationship between leukaemia incidence and electricity transmission equipment (*Br. J. Cancer*, 1989, **60**, 793).

However, I must point out that their statement that 'it was not possible to obtain direct measurements of field intensity . . . since . . . it was a condition of access to the Thames Cancer Registry data for the study that no contact would be made with study subjects or their kin' (p. 794) is perhaps misleading and indeed has been misconstrued by *The Times* (29 November 1989).

If I may clarify the Registry's access conditions: contact with patients or their relatives for interview *is* possible, provided that certain (albeit rather stringent) requirements are met. The requirements include obtaining the approval of an appropriate ethical committee and the permission of the patient's treating consultants and general practitioners. I must stress that restrictions to access are determined by the ethical committees and the medical practitioners who have responsibility for the care of each patient. The Thames Cancer Registry is always at pains to make its data available within this framework.

I would also point out that important interview studies that use the Thames Cancer Registry data as a starting point have been successfully undertaken (e.g. the widely publicised study of oral contraceptive use and breast cancer risk in young women in the *Lancet*, May 1989).

We welcome similar uses of our data.

Yours etc.,

H. Thornton-Jones
Registry Director,
The Thames Cancer Registry,
Clifton Avenue, Belmont,
Sutton, Surrey SM2 5PY, UK.